# Quantitative morphometric analysis of intrinsic and extrinsic skin ageing in individuals with Fitzpatrick skin types II–III

**DOI:** 10.1111/exd.14754

**Published:** 2023-02-03

**Authors:** Lydia Costello, Kirsty Goncalves, Paola De Los Santos Gomez, Amy Simpson, Victoria Maltman, Pamela Ritchie, Ryan Tasseff, Robert Isfort, Teresa Dicolandrea, Xingtao Wei, Arto Määttä, Iakowos Karakesisoglou, Ewa Markiewicz, Charles C. Bascom, Stefan Przyborski

**Affiliations:** ^1^ Department of Biosciences Durham University Durham UK; ^2^ Mason Business Centre, Procter and Gamble Ohio USA; ^3^ Hexis Lab Limited Newcastle upon Tyne UK; ^4^ Reprocell Europe Glasgow UK

**Keywords:** biometry, epidermis, skin ageing

## Abstract

Skin ageing is an intricate physiological process affected by intrinsic and extrinsic factors. There is a demand to understand how the skin changes with age and photoexposure in individuals with Fitzpatrick skin types I‐III due to accelerated photoageing and the risk of cutaneous malignancies. To assess the structural impact of intrinsic and extrinsic ageing, we analysed 14 skin parameters from the photoprotected buttock and photoexposed dorsal forearm of young and ageing females with Fitzpatrick skin types II‐III (*n* = 20) using histomorphic techniques. Whilst the minimum viable epidermis (E_min_) remained constant (*Q* > 0.05), the maximum viable epidermis (E_max_) was decreased by both age and photoexposure (*Q* ≤ 0.05), which suggests that differences in epidermal thickness are attributed to changes in the dermal‐epidermal junction (DEJ). Changes in E_max_ were not affected by epidermal cell proliferation. For the first time, we investigated the basal keratinocyte morphology with age and photoexposure. Basal keratinocytes had an increased cell size, cellular height and a more columnar phenotype in photoexposed sites of young and ageing individuals (*Q* ≤ 0.05), however no significant differences were observed with age. Some of the most striking changes were observed in the DEJ, and a decrease in the interdigitation index was observed with both age and photoexposure (*Q* ≤ 0.001), accompanied by a decreased height of rête ridges and dermal papilla. Interestingly, young photoexposed skin was comparable to ageing skin across many parameters, and we hypothesise that this is due to accelerated photoageing. This study highlights the importance of skin care education and photoprotection from an early age.

## 1 INTRODUCTION

Human skin ageing is a complex phenomenon associated with cumulative and progressive changes in skin structure that are attributed to both intrinsic and extrinsic factors. Intrinsic ageing refers to the genetically determined physiological decline that occurs over time, resulting in dry yet unblemished skin with fine lines and wrinkles. Extrinsic ageing is influenced by environmental factors, predominantly, sun exposure. Ultraviolet (UV) radiation is thought to contribute to 80% of facial ageing in individuals with Fitzpatrick skin types I‐III,^1^ and the clinical phenotype of photoageing includes coarse wrinkles, dyspigmentation and solar lentigines. The morphological impact of extrinsic ageing is superimposed upon intrinsic ageing, and different body sites can exhibit different rates of ageing depending on their environmental exposure. Photoageing is more prominent in lighter skin types due to decreased quantities of the photoprotective eumelanin and pheomelanin pigments.^2^

Understanding skin ageing is important for both geriatric and cosmetic dermatology. Ageing skin is associated with skin conditions such as pruritus, eczematous dermatoses and purpura. In addition to medical dermatoses, age‐related aesthetic changes in physical appearance can also have psychosocial effects such as reduced self‐esteem and altered self‐perception.^3^ Premature ageing can result as a consequence of cumulative sun exposure, and young individuals with Fitzpatrick skin types I–III often exhibit early signs of photoageing and develop fine lines and wrinkles in their twenties.^4^ Photodamage also increases the risk of cutaneous malignancies, and individuals with Fitzpatrick skin types I–III are more likely to be diagnosed with skin cancer due to their lower levels of innate photoprotection.^5^

Clinical tools such as SCINEXA have been developed to assess the extent of intrinsic and extrinsic skin ageing, however these are based on changes in skin topography and physical appearance such as wrinkles, elastosis and changes in pigmentation.^6^ The quantification of more intricate architectural changes to skin structure and cutaneous cells is important to define ageing biomarkers, identify therapeutic targets for preventative strategies and increase awareness about the importance of skin care education.

Studies investigating morphometric changes in human skin with age often focus on either intrinsic^7^, ^8^, ^9^, ^10^, ^11^ or extrinsic ageing.^12^, ^13^, ^14^ There is a paucity of information about how the skin tissue structure changes with both age and cumulative sun exposure, which is particularly important for individuals with Fitzpatrick skin types I–III due to the risk of photoageing and skin malignancies.

This study aimed to perform quantitative morphometry of skin morphology in photoprotected and photoexposed sites of young and ageing female individuals with Fitzpatrick skin types II‐III, to elucidate and quantify structural changes in skin. We focused on 14 parameters related to skin structure and demonstrate disparities between photoprotected and photoexposed sites, implying that intrinsic and extrinsic skin ageing have differential phenotypes. Our findings emphasise the importance of compliant photoprotective habits from an early age, and the development of advanced cosmetic products targeted towards a younger population.

## 2 METHODS

### 2.1 Study population

Full‐thickness 4 mm skin biopsies were obtained from the photoexposed dorsal forearm and photoprotected buttock of young, healthy, female volunteers (21–24 years, mean age 22.2 years, standard deviation (SD) 1.3 years, *n* = 10) and ageing, healthy, postmenopausal (one year since last menstrual cycle or by hysterectomy) female volunteers (61–65 years, mean age 62.9 years, SD 1.4 years, *n* = 10; Table S1). This sample size was selected based on a previous study that determined statistically significant changes through morphological assessment of histological materials.^15^ Our approach is further supported by other studies which report that significant histological changes can be gleamed from a sample size of *n* = 10 per demographic, which is either consistent or greater than examples of other histological analyses published to date.^7^, ^12^, ^16^, ^17^

All study subjects were non‐smokers, Fitzpatrick skin phototypes II and III and ageing individuals possessed moderate to severe photodamage in the photoexposed site, determined through assessment by a professional dermatologist. Strict exclusion criteria were applied to the study population such as prohibition of retinoid treatments, anti‐ageing treatments, anti‐acne treatment or hydroquinone‐containing treatments to the forearms in the 4 weeks prior to the study. Additionally, participants had no underlying chronic health conditions, either systemic or dermatological. Skin biopsies were collected by Procter and Gamble (P&G) under an IRB‐approved clinical protocol in compliance with local laws and regulations. Participants signed informed consent and were compensated for their participation in this study.

### 2.2 Processing of skin biopsies

Skin biopsies were fixed in 4% paraformaldehyde (Sigma‐Aldrich), serially dehydrated through a series of ethanol solutions (30%–100% v/v), then incubated in Histo‐Clear (Scientific Laboratory Supplies), and a 1:1 ratio of Histo‐Clear and paraffin wax (Thermo Fisher Scientific). Models were further incubated in paraffin wax prior to embedding in plastic moulds (Solmedia Ltd). Paraffin wax blocks were sectioned transversely at 5 μm using a microtome (Leica) and transferred onto charged microscope slides (Thermo Fisher Scientific) for analysis.

### 2.3 Histological staining and imaging

Sections were deparaffinised in Histo‐Clear and sequentially rehydrated from 100% ethanol to distilled water, before incubating in Mayer's haematoxylin (Sigma‐Aldrich) for 5 min. Sections were washed in distilled water, submerged in alkaline ethanol for 30 s and sequentially dehydrated to 95% ethanol. Samples were counter‐stained in eosin (Sigma‐Aldrich) for 30 s and then dehydrated to 100% ethanol. Sections were incubated twice in Histo‐Clear then mounted in Omnimount (Scientific Laboratory Supplies) prior to imaging. Samples were imaged using a Leica microscope, and images were captured using the Leica EZ software. Four sections per skin biopsy (*n* = 40 skin biopsies, *n* = 160 sections) were stained and imaged along their entire length at 20× magnification, and images were stitched together using Fiji software to visualise the complete skin section.^18^ These images were used for quantification of epidermal thickness, interdigitation index, rête ridge morphology and dermal papilla morphology (Figure S1A,D–F).

### 2.4 Immunofluorescence staining and imaging

Sections were deparaffinised in Histo‐Clear and sequentially rehydrated from 100% ethanol to phosphate‐buffered saline (PBS). Antigen retrieval was performed by incubating samples in pH 6 citrate buffer (Sigma‐Aldrich) at 95°C for 20 min. Samples were blocked and permeabilised for 1 h in a blocking buffer of 20% neonatal calf serum (Thermo Fisher Scientific) in 0.4% Triton X‐100 in PBS. Primary antibodies (cytokeratin 14 ab7800, cytokeratin 10 ab76318, Ki67 ab16667; Abcam) at a 1:100 dilution in blocking buffer were incubated with samples at 4°C overnight. Samples were washed three times in PBS and incubated with secondary antibodies (donkey anti‐mouse Alexa Fluor^®^ 488 A21202; donkey anti‐rabbit Alexa Fluor^®^ 594 A21207, donkey anti‐rabbit Alexa Fluor^®^ 488 A‐21206; Thermo Fisher Scientific) at a 1:1000 dilution in blocking buffer for 1 h at room temperature. Samples were washed three times in PBS before mounting in Vectashield Hardset with DAPI mounting medium (Vector Laboratories). The fluorescent images were captured using a Zeiss 880 confocal microscope (Zeiss) with Zen software. Two sections per skin biopsy (*n* = 40 skin biopsies; *n* = 80 sections) were stained, and three random images were taken per section at 40x magnification for biometrics quantification (*n* = 240 images total). These images were used to measure basal keratinocyte morphology and quantify the number of Ki67‐positive cells (Figure S1B,C).

### 2.5 Blinding and randomising of images

To remove any unconscious bias, the images were blinded and randomised by an independent individual. The images were given a random 3‐digit code (001‐160 for histological images and 001‐240 for immunofluorescence images), which were de‐coded for statistical analysis once the measurements were complete.

### 2.6 Measurement acquisition

Measurements were taken by skin scientists possessing either a Master's degree or Doctorate in the field, who are experienced at measuring these parameters. To remove interobserver variation and ensure consistency, the same individual measured each distinct parameter across all randomised and blinded samples. All annotated images were then saved and checked by an independent observer for quality control purposes prior to deblinding. For Ki67 expression determined by immunofluorescence, two individuals made the measurements and then quality checked the other's measurements. No significant difference in the measurement of Ki67 between the two individuals was observed.

### 2.7 Biometrics analysis of skin parameters

A detailed overview of the biometric measurements methodology using histological and immunofluorescence images is presented in Figure S1.

### 2.8 Epidermal thickness

The minimum thickness of the viable epidermis (E_min_) was measured using the suprapapillary epidermis, from the top of the dermal papillae to the top of the *stratum granulosum* in H&E‐stained skin samples using the line tool in the Fiji software (Figure S1A). The maximum thickness of the viable epidermis (E_max_) was measured from the bottom of the rête ridges to the top of the *stratum granulosum* (Figure S1A). Measurements were taken at regular intervals along the entire length of the blinded and randomised skin biopsy images. Four sections per skin sample were used (*n* = 40 skin biopsies, *n* = 160 sections, *n* = 160 stitched images).

### 2.9 Epidermal proliferation

Epidermal proliferation was quantified using images of Ki67‐stained skin sections. The multipoint tool in the Fiji software was used to count the number of Ki67‐positive nuclei and total number of DAPI‐stained nuclei in blinded and randomised images, which were used to calculate the percentage of Ki67‐positive cells in the epidermis (Figure S1B). Two sections per skin sample were stained, and three random images were taken per section (*n* = 40 skin biopsies, *n* = 80 sections, *n* = 240 images).

### 2.10 Basal keratinocyte morphology

Skin sections stained with cytokeratin 14 and cytokeratin 10 were used to measure the area, height and width of the basal keratinocytes. All basal keratinocytes that were K14‐positive and in contact with the basement membrane within an image were measured. The polygon and line tools in the Fiji software were used to annotate and measure the parameters in the blinded and randomised images (Figure S1C). Two sections per skin sample were stained, and three random images were taken per section (*n* = 40 skin biopsies, *n* = 80 sections, *n* = 240 images).

### 2.11 Interdigitation index

The interdigitation index of the skin samples was determined using the Skin Tools Image J Macro according to the manufacturer's instructions,^19^ which was developed from the original manual protocol.^7^ Briefly, a mask image of the entire epidermis from blinded and randomised skin biopsy sections was created in Image J, and exported into the Skin Tools Macro to calculate the interdigitation index across 15 segments of the section (Figure S1D). Any segments containing artefacts such as hair follicles that could skew the data were omitted. Four sections per skin sample were used (*n* = 40 skin biopsies, *n* = 160 sections, *n* = 160 stitched images).

### 2.12 Rête ridges and dermal papilla morphology

The polygon and line tools in the Fiji software were used to annotate and measure the area, height and width of all rête ridges and dermal papillae within the blinded and randomised H&E‐stained images (Figure S1E,F). Four sections per skin sample were used (*n* = 40 skin biopsies, *n* = 160 sections, *n* = 160 stitched images).

### 2.13 Statistical analysis

For all skin parameters, the mean of the measurements taken from each image was calculated to avoid data skewing, for example due to different numbers of rête ridges and dermal papilla between samples (*n* = 4 stitched images per skin biopsy for H&E‐stained images and *n* = 6 images per skin biopsy for immunofluorescence images). The means from each skin biopsy were used to calculate the demographic results, which are expressed as mean ± standard error of the mean (SEM). Data is presented as a bar chart based on measurements obtained from 40 skin biopsies. A linear mixed‐effects model was fitted to each parameter with the subject as the random effect and age and photoexposing conditions as the fixed effects. Treating the subject as the random effect in the model was to account for the correlation between photoprotected and photoexposed samples from the same subject, and this also allows us to draw conclusions on the population those subjects represent. The Benjamini‐Hochberg (BH) method^20^ was used to adjust the *p*‐values from all comparisons, and the adjusted *p*‐values, commonly called q‐values, are to control the false discovery rate (FDR). All statistical analyses were performed using the R (version 4.2.1).^21^ Differences between the groups were considered significant when *Q* ≤ 0.05, and the significance is depicted graphically for each data set where *Q ≤ 0.05, **Q ≤ 0.01, ***Q ≤ 0.001, ****Q ≤ 0.0001, *****Q ≤ 0.00001, not significant (ns) *Q >* 0.05.

## 3 RESULTS

### 3.1 Study population

Two discrete age categories were selected for participants (young: 21–24 years; ageing: 61–65 years) and biopsies were obtained from both photoprotected and photoexposed sites to evaluate structural changes with age and photoexposure. The participants were all female with Fitzpatrick skin phototypes II and III (young: 10% type II and 90% type III; ageing: 20% type II and 80% type III), to minimise sex‐specific and phototype‐specific variation in photoageing.

### 3.2 Maximum epidermal thickness is affected by age and photoexposure

**FIGURE 1:**
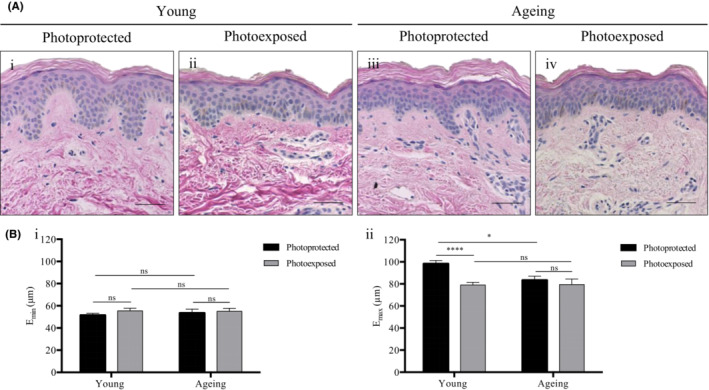
E_min_ and E_max_ are differentially affected by age and photoexposure. (A) Representative histological micrographs of human skin obtained from (i, iii) photoprotected buttock sites and (ii, iv) photoexposed dorsal forearm sites of (i, ii) a young 24 year old donor, and (iii, iv) an ageing 64 year old donor. Scale bars: 50 μm. (B) (i) E_min_ and (ii) E_max_ of the viable epidermis from photoprotected and photoexposed sites in young and aged subjects of Fitzpatrick II‐III skin types (*n* = 10 donors per category). Mean values ± SEM are displayed, *Q ≤ 0.05, ****Q ≤ 0.0001, ns *Q >* 0.05.

The thickness of the viable epidermis can be measured using the suprapapillary epidermis alone or whole epidermis including the rête ridges, which are denoted as E_min_ and E_max_ respectively (Figure S1A). Previous studies have measured E_min_,^13^, ^16^, ^22^, ^23^ E_max_ ^24^, ^25^ or both.^9^, ^26^ Histological analysis of human skin in Figure 1A demonstrated differences in the DEJ with age and photoexposure, therefore, we decided to measure both E_min_ and E_max_ in our study.

The suprapapillary epidermal thickness, E_min_, did not change with age or photoexposure (young; buttock: 51.6 ± 1.7 μm, forearm: 55.4 ± 2.4 μm, ageing; buttock: 53.5 ± 3.4 μm, forearm: 55.0 ± 2.8 μm) (*Q* > 0.05) (Figure 1Bi). In contrast, the E_max_ was affected by both age and photoexposure (young; buttock: 98.3 ± 2.9 μm, forearm: 79.0 ± 2.4 μm, ageing; buttock: 83.4 ± 3.6 μm, forearm: 79.4 ± 5.0 μm) (Figure 1Bii). In young individuals, there was a significant 19.6% decrease in E_max_ with photoexposure (*Q* ≤ 0.0001) however no significant differences were observed between sites in ageing individuals (*Q* > 0.05). Interestingly, the E_max_ was comparable between young photoexposed and ageing photoprotected and photoexposed skin, which indicates that young photoexposed sites could exhibit accelerated extrinsic photoageing. Whilst no significant differences were observed between photoexposed sites with age, there was a 15.2% decrease in E_max_ with age in photoprotected sites (*Q* ≤ 0.05), which suggests that age‐related changes are more gradual with intrinsic ageing in the photoprotected buttock.

### 3.3 Epidermal proliferation is not affected by age or photoexposure

To investigate whether changes in E_max_ were driven by differences in keratinocyte proliferation, we measured the expression of Ki67, a classical marker of cellular proliferation.

**FIGURE 2:**
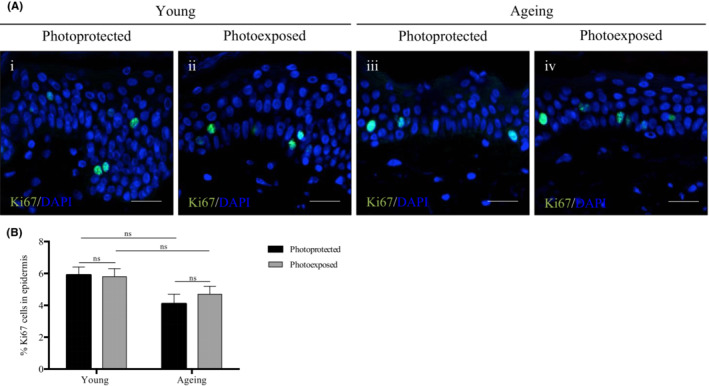
Epidermal proliferation not affected by age in photoexposed and photoprotected sites. (A) Representative immunofluorescence images of human skin obtained from (i, iii) photoprotected buttock sites and (ii, iv) photoexposed dorsal forearm sites of (i, ii) a young 22 year old donor, and (iii, iv) an ageing 64 year old donor. (i–iv) Immunolabelling of Ki67 and DAPI nuclei staining. Scale bars: 50 μm. (B) The % Ki67 of the epidermis from photoprotected and photoexposed sites in young and aged individuals with Fitzpatrick II‐III skin types (*n* = 10 donors per category). Mean values ± SEM are displayed, ns *Q >* 0.05.

As demonstrated in Figure 2A, Ki67‐positive cells were located in the basal and suprabasal layers of the epidermis. No significant differences in the percentage of Ki67‐positive cells within the epidermis were observed with age or photoexposure in young and ageing individuals (*Q* > 0.05). These results suggest that the observed changes in E_max_ are not affected by epidermal proliferation.

### 3.4 Basal keratinocyte morphology is altered by photoexposure but not age

Characterisation of cutaneous cell geometry within the *stratum spinosum, stratum granulosum* and *stratum corneum* with age has been well‐established,^9^, ^10^, ^12^, ^27^, ^28^ and in this study we determined how basal keratinocyte morphology is altered with both age and photoexposure. This is particularly interesting as basal keratinocytes are in direct contact with the dermal‐epidermal junction, which undergoes distinct morphological changes, as described in Figure 4.

**FIGURE 3:**
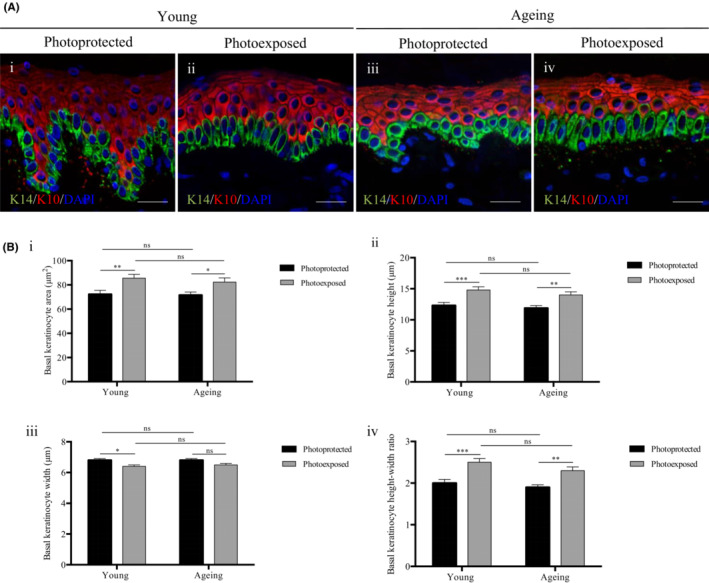
Photoexposure affects basal keratinocyte morphology. (A) Representative immunofluorescence images of human skin obtained from (i, iii) photoprotected buttock sites and (ii, iv) photoexposed dorsal forearm sites of (i, ii) a young 22 year old donor, and (iii, iv) an ageing 64 year old donor. (i–iv) Immunolabelling of cytokeratin 14 and cytokeratin 10 and DAPI nuclei staining. Scale bars: 25 μm. (B) The (i) area, (ii) height, (iii) width and (iv) height‐width ratio of basal keratinocytes from photoprotected and photoexposed sites in young and aged subjects of Fitzpatrick Scale II‐III (*n* = 10 donors per category). Mean values ± SEM are displayed, *Q ≤ 0.05, **Q ≤ 0.01, ***Q ≤ 0.001, ns *Q >* 0.05.

Human skin has a well‐organised *stratum basale* regardless of age or photoexposure, however some disparities in basal keratinocyte structure were observed using immunofluorescence analysis (Figure 3A). These morphological differences were quantified through parameters such as area, height and width.

In young and ageing individuals, there was a significant 18.4% and 15.1% increase in basal keratinocyte area in photoexposed sites respectively (*Q* ≤ 0.05), however no differences were observed with age (Figure 3Bi). We analysed whether this increase in cell size with photoexposure was attributed to changes in cellular morphology. Quantification of cell height confirmed this, as the height of basal keratinocytes was significantly increased in photoexposed sites by 20.3% and 17.6% in young and ageing individuals respectively (*Q* ≤ 0.01) (Figure 3Bii). The basal keratinocyte width was decreased with photoexposure in young individuals, but to a lesser extent (*Q* ≤ 0.05) (Figure 3Biii). This suggests that the increase in cell size with photoexposure is attributed to increased height of basal keratinocytes, and the height‐width ratio was calculated to provide an indication of changes in cell shape. In both young and ageing individuals, the height‐width ratio of basal keratinocytes was significantly increased in photoexposed sites, which is indicative of a more columnar structure and reflects the morphology observed in Figure 3A (young; buttock: 2.0 ± 0.1 μm, forearm: 2.5 ± 0.1 μm, ageing; buttock: 1.9 ± 0.1 μm, forearm: 2.3 ± 0.1 μm) (*Q* ≤ 0.01) (Figure 3Biv).

To summarise, the morphology of basal keratinocytes is affected by photoexposure, with an increase in cell size, increased height and a more columnar morphology (*Q* ≤ 0.05). No significant differences in basal keratinocyte morphology were observed with age (*Q* > 0.05).

### 3.5 Dermal‐epidermal junction characteristics are influenced by age and photoexposure

Histological analysis of human skin and disparities in E_max_ measurements identified in Figure 1 suggested that the viable epidermal thickness is affected by changes in the DEJ. To determine age‐ and photoexposure‐related differences, we quantified the interdigitation index and the area, height and width of the rête ridges and dermal papilla.

### 3.6 Interdigitation index

The interdigitation index is a compelling measure of dermal‐epidermal junction undulations, first described by Timár et al., 2000 and used in many dermatological studies.^7^, ^9^, ^12^, ^14^, ^26^, ^29^ In this study, we demonstrate changes with both age and photoexposure.

**FIGURE 4:**
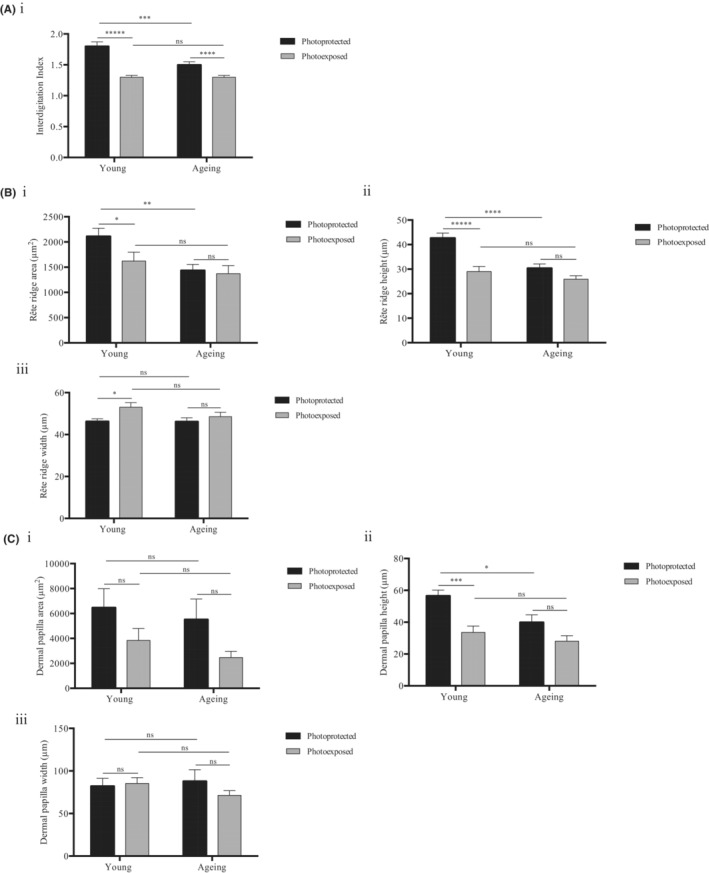
Age‐ and photoexposure‐related disparities in the DEJ. (A) The interdigitation index, (B) The (i) area, (ii) height and (iii) width of rête ridges, and (C) The (i) area, (ii) height and (iii) width of dermal papilla from photoprotected and photoexposed sites in young and aged subjects of Fitzpatrick II‐III phototype (*n* = 10 donors per category). Mean values ± SEM are displayed, *Q ≤ 0.05, **Q ≤ 0.01, ***Q ≤ 0.001, ****Q ≤ 0.0001, *****Q ≤ 0.00001, ns *Q >* 0.05.

The interdigitation index was significantly decreased with photoexposure by 27.8% and 13.3% in young and ageing individuals respectively (young; buttock: 1.8 ± 0.07 μm, forearm: 1.3 ± 0.03 μm, ageing; buttock: 1.5 ± 0.05 μm, forearm: 1.3 ± 0.03 μm) (*Q* ≤ 0.0001, Figure 4A). Age‐related differences were also observed, as the interdigitation index of photoprotected sites decreased with age by 16.7% (young: 1.8 ± 0.07 μm; ageing: 1.5 ± 0.05 μm) (*Q* ≤ 0.001). Interestingly, the interdigitation index was comparable between photoexposed sites in young and ageing individuals (*Q* > 0.05). We hypothesise that extrinsic ageing accelerates the characteristic flattening of the dermal‐epidermal junction, with a more gradual change in intrinsically aged sites.

We determined how the flattening of the dermal‐epidermal junction captured by the interdigitation index correlates with morphological changes in rête ridges and dermal papilla.

### 3.7 Rête ridges

In young individuals, there was a significant 23.1% decrease in rête ridge area with photoexposure (*Q* ≤ 0.05) however no significant differences were observed between photoprotected and photoexposed sites in ageing individuals (*Q* > 0.05) (Figure 4Bi). In photoprotected sites, rête ridge area significantly decreased by 31.9% with age (*Q* ≤ 0.01) however no significant changes in rête ridge area were observed with age in photoexposed sites (*Q* > 0.05, Figure 4Bi).

Rête ridge height demonstrated a similar trend to rête ridge area, as there was a significant 32.1% decrease with photoexposure in young individuals (*Q* ≤ 0.0001), however no significant differences were observed between photoprotected and photoexposed sites in ageing individuals (*Q* > 0.05) (Figure 4Bii). Similarly, in the photoprotected buttock, the rête ridge height decreased by 28.8% with age (*Q* ≤ 0.0001), whereas no significant change was observed in the photoexposed forearm with age (*Q* > 0.05) (Figure 4Bii).

Conversely to the rête ridge height, the width was significantly increased by 14.7% in the photoexposed forearm of young individuals, compared to the photoprotected buttock (forearm: 53.1 ± 2.2 μm, buttock 46.3 ± 1.2 μm) (*Q* ≤ 0.05). There was no significant difference in the rête ridge width with photoexposure in ageing individuals, and no significant changes were observed with age in either site (*Q* > 0.05, Figure 4Biii).

### 3.8 Dermal papilla

No significant changes were observed in dermal papilla area with age or photoexposure (*Q* > 0.05, Figure 4Ci). Interestingly, the dermal papilla area was more variable than rête ridges at both sites of young and ageing individuals.

In young individuals, there was a significant 40.5% decrease in dermal papilla height with photoexposure (*Q* ≤ 0.001), whereas no significant differences were observed with photoexposure in ageing individuals (*Q* > 0.05). In photoprotected sites, there was a significant 29.5% decrease in dermal papilla height with age (*Q* ≤ 0.05), which suggests that there is a progressive change in the DEJ with intrinsic ageing. Consistent with the E_max_, rête ridges and interdigitation index, no significant differences were observed in photoexposed sites of young and ageing individuals (*Q* > 0.05), which supports the hypothesis of accelerated photoageing at extrinsic sites (Figure 4Cii). No significant difference in the dermal papilla width was observed with age or photoexposure (*Q* > 0.05).

To summarise, a decreased interdigitation index and decreased height of rete ridges and dermal papilla was observed with age and photoexposure, which is indicative of the flattening of the DEJ. Strikingly, no significant differences were observed between young and ageing photoexposed skin for any of the DEJ parameters.

### 3.9 Summary of findings

**TABLE 1:** Summary of the histomorphic measurements of young and ageing photoprotected and photoexposed skin.

	Young (21–24 years)		Ageing (61–65 years)	
	Photoprotected ± SEM	Photoexposed ± SEM	Photoprotected ± SEM	Photoexposed ± SEM
E_min_ (μm)	51.6 ± 1.7	55.4 ± 2.4	53.5 ± 3.4	55.0 ± 2.8
E_max_ (μm)	98.3 ± 2.9	79.0 ± 2.4	83.4 ± 3.6	79.4 ± 5.0
Epidermal proliferation (% Ki67‐positive cells)	5.9 ± 0.5	5.8 ± 0.5	4.1 ± 0.6	4.7 ± 0.5
Basal keratinocytes				
Area (μm^2^)	72.3 ± 3.2	85.6 ± 3.2	71.6 ± 2.5	82.4 ± 3.4
Height (μm)	12.3 ± 0.5	14.8 ± 0.5	11.9 ± 0.4	14.0 ± 0.5
Width (μm)	6.8 ± 0.1	6.4 ± 0.1	6.8 ± 0.1	6.5 ± 0.1
Height‐width ratio	2.0 ± 0.1	2.5 ± 0.1	1.9 ± 0.1	2.3 ± 0.1
Interdigitation index	1.8 ± 0.07	1.3 ± 0.03	1.5 ± 0.05	1.3 ± 0.03
Rête ridges				
Area (μm^2^)	2110.5 ± 161.5	1622.3 ± 175.8	1437.4 ± 117.7	1371.9 ± 158.4
Height (μm)	42.7 ± 2.0	29.0 ± 2.1	30.4 ± 1.7	25.9 ± 1.4
Width (μm)	46.3 ± 1.2	53.1 ± 2.2	46.2 ± 1.8	48.6 ± 2.1
Dermal papilla				
Area (μm^2^)	6474.2 ± 1514.9	3842.9 ± 954.7	5524.1 ± 1642.2	2473.4 ± 492.2
Height (μm)	56.6 ± 3.6	33.6 ± 4.0	39.9 ± 4.7	28.0 ± 3.5
Width (μm)	82.2 ± 9.1	85.3 ± 6.7	87.9 ± 13.5	71.3 ± 5.5

## 4 DISCUSSION

The deleterious effect of cumulative environmental exposure, particularly UV radiation, in individuals with Fitzpatrick skin types I‐III is widely acknowledged, and there is a demand to further characterise how the skin changes with both age and photoexposure. Many studies focus on intrinsic^7^, ^10^, ^11^, ^30^ or extrinsic skin ageing^11^, ^12^, ^13^, ^14^ and few studies investigate cutaneous changes with both age and photoexposure in Fitzpatrick skin types I‐III individuals.^22^, ^26^ In addition, most studies recruit both male and female participants despite the well‐documented sex‐specific differences in skin structure and function.^31^ Using quantitative histometric techniques, we analysed 14 parameters relating to cellular and structural skin disparities with both age and photoexposure in female individuals with Fitzpatrick skin types II‐III, and our findings are summarised in Figure S2.

A wide variety of existing studies have aimed to elucidate structural changes in skin with age, however their limitations include: small number of parameters measured, mixed sex participants, varied Fitzpatrick skin types and a sample size of less than 10 individuals. In this study we have aimed to improve these limitations by focusing our target demographic to female Fitzpatrick I–III participants to control some of the aforementioned variables. Furthermore, we selected a sample size of 10 individuals, as statistical significance has been determined from a similar sample size in other histometric studies.^7^, ^12^, ^16^, ^17^ It is worth noting that a smaller sample size may be a limitation of these studies and increasing the population sample size may further strengthen the findings.

We have taken sufficient steps to mitigate any sample bias such as the diligent analysis of up to 160 micrographs (four slides per individual, 10 individuals per demographic, four demographics), stringent exclusion criteria and sample blinding. All of which will have had a large impact on minimising anomalous measurements and sectioning/sampling errors. Thus providing a robust, reliable and accurate representation of histological changes in the skin samples.

Interestingly, the most structural differences were observed between photoprotected and photoexposed skin in young individuals, with minimal differences between young photoexposed and ageing photoexposed skin (Figure S2). This supports the hypothesis that young forearm skin exhibits accelerated photoageing, and we hypothesise that the forearm skin reaches an “ageing threshold”, which is maintained with age.

### 4.1 Epidermal thickness and proliferation

There are discrepancies in the literature regarding the methodologies used to measure epidermal thickness. Some studies report the thickness of the viable suprapapillary epidermis (E_min_),^13^, ^16^, ^22^, ^23^ whereas other studies do not distinguish between the suprapapillary epidermis and regions including rête ridges (E_max_).^24^, ^25^ In our study, we investigated how both the minimum and maximum epidermal thickness were affected by age and photoexposure in individuals with Fitzpatrick skin types II‐III.

Our results suggest that E_min_ does not change with age or photoexposure, whereas E_max_ is affected by both age and photoexposure. This implies that the observed changes in the epidermal thickness are attributed to the flattening of the dermal‐epidermal junction, rather than decreased thickness of the viable suprapapillary plate. This is in agreement with studies using in vivo techniques such as harmonic generation microscopy (HGM) and optical coherence tomography (OCT).^9^, ^26^ The differences between E_min_ and E_max_ highlight the need for consistent methodologies of measuring epidermal thickness for comparison between studies.

There are some inconsistencies in the literature regarding the expression of Ki67, as studies report an increase,^32^ decrease^33^ or no significant difference^34^ in Ki67 expression with age. Chronic, daily exposure to UV is thought to increase Ki67 expression in photoprotected skin under experimental conditions,^35^ however few studies have compared epidermal proliferation in photoexposed and photoprotected sites. Interestingly, we did not observe any difference in the percentage of Ki67‐positive cells in the epidermis with age or photoexposure.

### 4.2 Basal keratinocyte morphology

Skin ageing is associated with increased corneocyte size,^27^ a consistent cell size within the *stratum granulosum,* ^9^, ^10^, ^12^ and increased keratinocyte size within the *stratum spinosum* in photoexposed sites.^28^ Some studies have investigated morphological changes in basal keratinocytes with age, however to our knowledge, we describe the first study investigating basal keratinocyte size and geometry across photoprotected and photoexposed sites in young and ageing individuals.

We identified a significant increase in basal keratinocyte area in photoexposed sites in both young and ageing individuals. However, no significant differences were observed with age. This is in disagreement with previous findings, as age‐related increases in basal keratinocyte size have been reported in photoexposed sites of Asian individuals and photoprotected sites of Caucasian individuals,^9^, ^10^ however changes with photoexposure have not been previously investigated. In addition, basal keratinocytes in photoexposed sites exhibited a more columnar geometry with a significant increase in cellular height, decreased width and increased height‐width ratio. We propose that altered basal keratinocyte morphology could be a biomarker of photoageing, however further studies are required to elucidate the link between basal keratinocyte structure and functionality.

### 4.3 Dermal‐epidermal junction

The flattening of the dermal‐epidermal junction during ageing has many physiological consequences. The surface area between the epidermis and dermis is thought to decrease by approximately 35%,^36^ which increases skin fragility by reducing both resistance to shear stress and the exchange of oxygen, nutrients and signalling molecules to the asvascular epidermis.

The interdigitation index has been reported to decrease with age in photoprotected sites of both Asian and Caucasian populations,^7^, ^9^, ^10^ which is consistent our findings. In contrast to other studies, we also investigated how the interdigitation index changed with photoexposure, and observed a significant decrease in photoexposed sites in both young and ageing individuals. The most striking difference was observed in the young individuals, and we hypothesise that the combined effect of intrinsic and extrinsic ageing in the forearm accelerates the ageing phenotype, and alterations in the DEJ could be some of the first structural changes that occur during photoageing. Interestingly, there was no significant difference between photoexposed sites in young and ageing individuals, which suggests that DEJ could reach an ageing threshold at an index of 1.3.

We also investigated how the geometry of the dermal papilla and rête ridges change with age and photoexposure. The height of these structures was decreased with age in intrinsic sites, similar to other findings in the literature studying Asian and Caucasian populations.^9^, ^10^, ^37^ The effect of photoexposure has not been previously delineated, and we also report a decreased height of rête ridges and dermal papilla in photoexposed sites.

In all DEJ measurements, striking differences were observed between photoexposed and photoprotected sites of young individuals, however the photoexposed sites did not change significantly with age, which supports the accelerated ageing hypothesis.

## 5 CONCLUSIONS

Differential changes in skin structure have been identified with age and photoexposure and are consistent with previous transcriptomic analyses.^38^ This study demonstrates that early morphological signs of ageing such as flattening of the dermal‐epidermal junction can be observed in photoexposed skin of young individuals in their early twenties. This demonstrates the importance of photoprotective habits such as wearing daily sunscreen with a high sun protection factor, checking the UV index to limit sun exposure at peak hours and wearing protective clothing. Due to the early signs of ageing in photoexposed sites, the use of dermocosmetic products that contain anti‐ageing actives such as retinoids could be beneficial at a younger age to attenuate the age‐related alterations.

## AUTHOR CONTRIBUTIONS

LC, SP, CB, AM, IK and EM helped develop the initial concepts behind the study. LC was largely responsible for the experimental plan and initial drafting the manuscript with significant input by EM. CB, TD, RT, RI, EM, AM and IK provided valuable feedback on parameters of interest. LC, KG, PD, AS and VM contributed to the quantitative measurements outlined in this study. PR provided technical support and XW performed statistical analyses. All co‐authors reviewed and approved the final manuscript. SP is the Principal Investigator and corresponding author.

## CONFLICT OF INTEREST STATEMENT

The authors have declared no conflicting interests.

## Supporting information


**Figure S1** Biometric methodology used to quantify skin parameters.
**Figure S2** Age‐ and photoexposure‐related changes in human skin morphology. The structure of human skin in female individuals with Fitzpatrick Scale II‐III phototypes is differentially affected by age and photoexposure, and architectural differences in epidermal thickness and proliferation, basal keratinocyte morphology and DEJ characteristics are observed.
**Table S1** Study population.

## Data Availability

The data that support the findings of this study are available from the corresponding author upon reasonable request.
