# Supplementary material for: Quantitative morphometric analysis of intrinsic and extrinsic skin ageing in individuals with Fitzpatrick skin types II–III
Source: Exp Dermatol. 2023 Feb 3;32(5):620–31. doi: 10.1111/exd.14754 (PMC10947487; doi:10.1111/exd.14754)
Supplement: Supplementary file 1 — Figure S1 Biometric methodology used to quantify skin parameters. Figure S2 Age‐ and photoexposure‐related changes in human skin morphology. The structure of human skin in female individuals with Fitzpatrick Scale II‐III phototypes is differentially affected by age and photoexposure, and architectural differences in epidermal thickness and proliferation, basal keratinocyte morphology and DEJ characteristics are observed. Table S1 Study population. [file EXD-32-620-s001.pptx]

## Slide 1
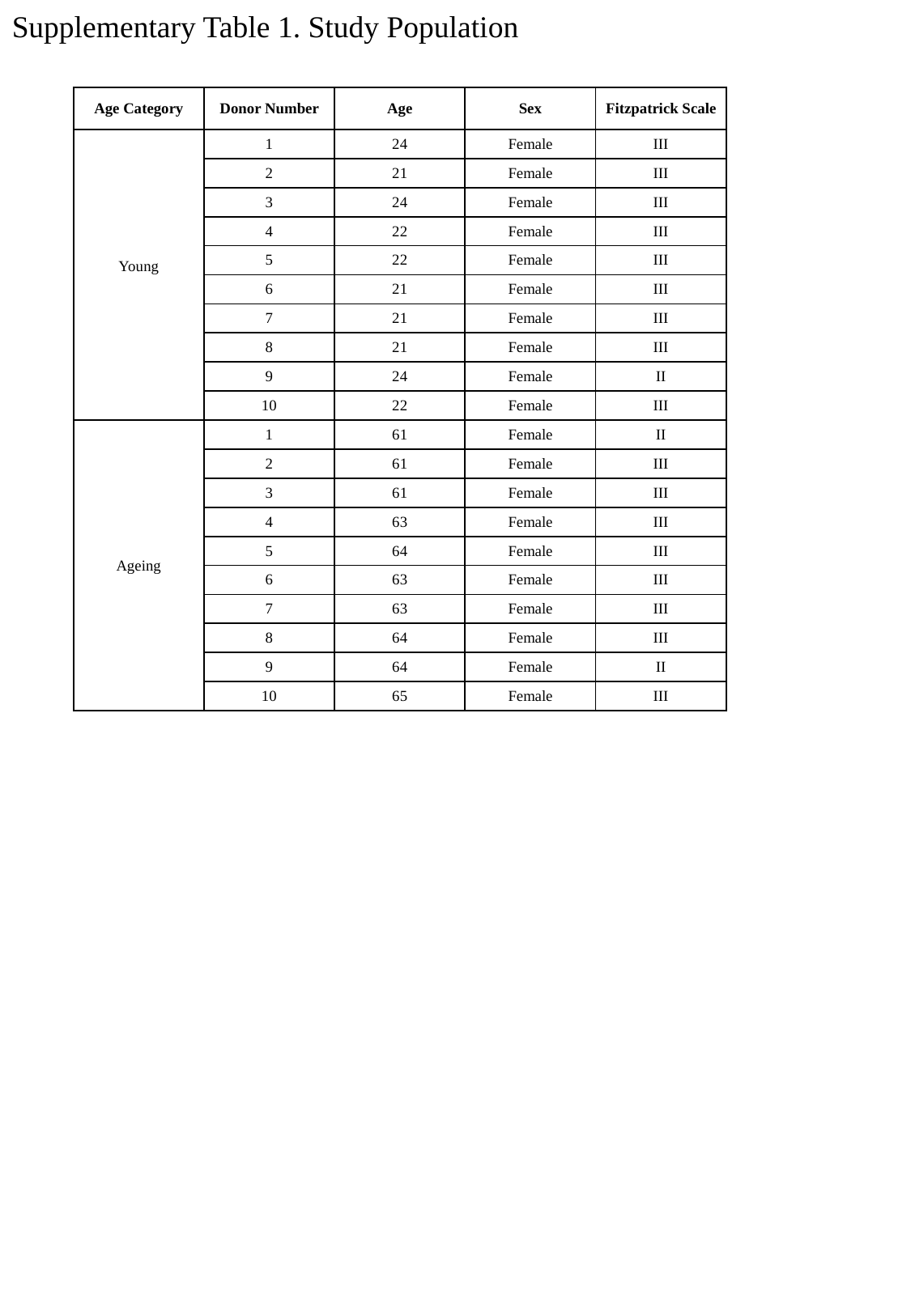

Supplementary Table 1. Study Population
| Age Category | Donor Number | Age | Sex | Fitzpatrick Scale |
| --- | --- | --- | --- | --- |
| Young | 1 | 24 | Female | III |
| | 2 | 21 | Female | III |
| | 3 | 24 | Female | III |
| | 4 | 22 | Female | III |
| | 5 | 22 | Female | III |
| | 6 | 21 | Female | III |
| | 7 | 21 | Female | III |
| | 8 | 21 | Female | III |
| | 9 | 24 | Female | II |
| | 10 | 22 | Female | III |
| Ageing | 1 | 61 | Female | II |
| | 2 | 61 | Female | III |
| | 3 | 61 | Female | III |
| | 4 | 63 | Female | III |
| | 5 | 64 | Female | III |
| | 6 | 63 | Female | III |
| | 7 | 63 | Female | III |
| | 8 | 64 | Female | III |
| | 9 | 64 | Female | II |
| | 10 | 65 | Female | III |

## Slide 2
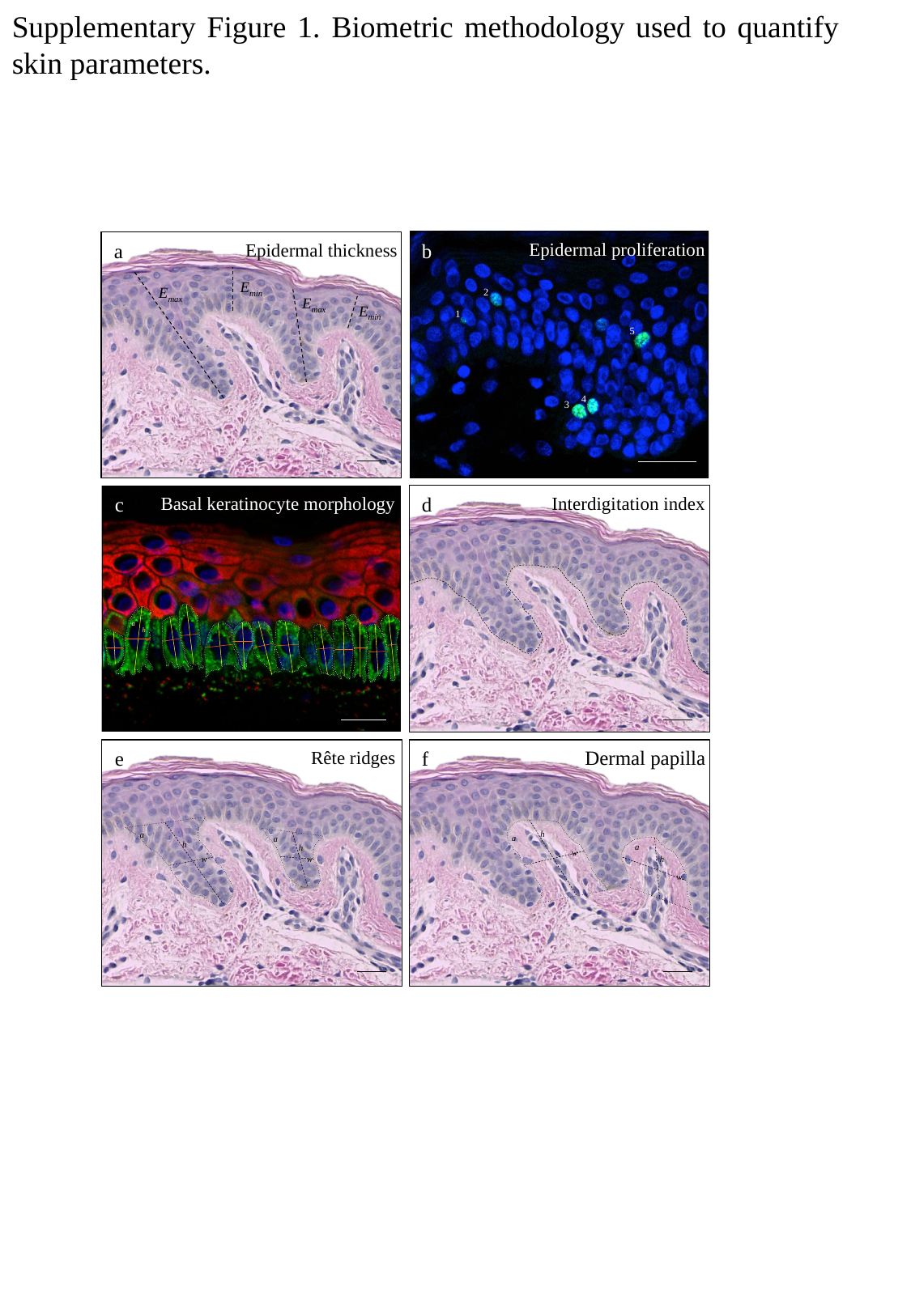

Supplementary Figure 1. Biometric methodology used to quantify skin parameters.
Epidermal proliferation
a
Epidermal thickness
Emin
Emax
Emax
Emin
b
2
1
5
4
3
Basal keratinocyte morphology
Interdigitation index
d
c
a
h
w
Dermal papilla
e
Rête ridges
f
h
a
a
a
h
a
h
w
w
h
w
w

## Slide 3
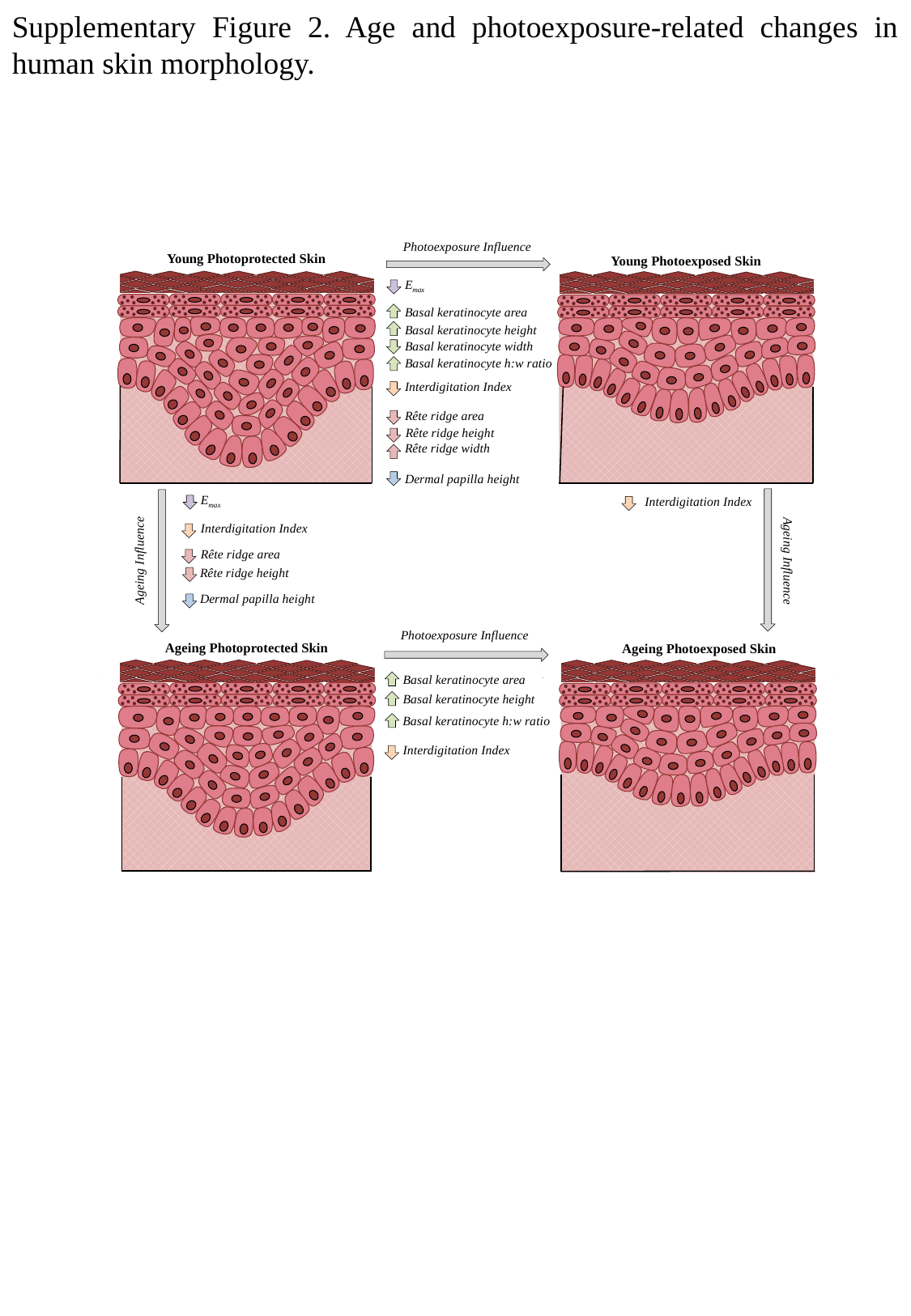

Supplementary Figure 2. Age and photoexposure-related changes in human skin morphology.
Photoexposure Influence
Young Photoprotected Skin
Young Photoexposed Skin
Emax
Basal keratinocyte area
Basal keratinocyte height
Basal keratinocyte width
Basal keratinocyte h:w ratio
Interdigitation Index
Rête ridge area
Rête ridge height
Rête ridge width
Dermal papilla height
Emax
Interdigitation Index
Interdigitation Index
Rête ridge area
Ageing Influence
Ageing Influence
Rête ridge height
Dermal papilla height
Photoexposure Influence
Ageing Photoprotected Skin
Ageing Photoexposed Skin
Basal keratinocyte area
Basal keratinocyte height
Basal keratinocyte h:w ratio
Interdigitation Index
